# A 900 MHz, Wide-Input Range, High-Efficiency, Differential CMOS Rectifier for Ambient Wireless Powering

**DOI:** 10.3390/s22030974

**Published:** 2022-01-27

**Authors:** Abdulaziz Alhoshany

**Affiliations:** Department of Electrical Engineering, College of Engineering, Qassim University, Al-Malida, Buraydah 52571, Saudi Arabia; a.alhoshany@qec.edu.sa or ahoshany@qu.edu.sa

**Keywords:** RF-to-DC power converter, CMOS rectifiers, wide input range, efficiency, energy harvesting, wireless power transfer

## Abstract

This paper presents a wide dynamic-range CMOS rectifier with high efficiency and high sensitivity for RF energy harvesting. A new adaptive-biasing scheme is implemented using stacking diodes with dynamic threshold voltage to mitigate the reverse-leakage current of the NMOS rectifying devices at high RF power levels. The proposed design employs the adaptive-biasing technique to control the conduction of the PMOS rectifying devices with self-bulk biasing of the feedback diodes to minimize the leakage current. The proposed novel techniques extend the dynamic range of the RF-to-DC power converter with high efficiency, which is 17 times better than a conventional cross-coupled rectifier. The prototype is implemented using a standard 65 nm CMOS technology and occupies a 0.0093 mm^2^ active area. The proposed design achieves a peak power conversion efficiency (peak PCE) of 73%, −18.8 dBm 1-V sensitivity, and a superb dynamic range of 17.3 dB with efficiency greater than 80% of its peak PCE, which outperforms the state-of-the-art RF CMOS rectifiers, when operating at UHF 900 MHz with a 100-KΩ load.

## 1. Introduction

The advancement in wireless power transfer (WPT) techniques and integrated circuits technology has enabled an evolution of miniaturized battery-less electronic devices such as biomedical implants, wireless sensors, and radio frequency identifications (RFIDs) [1,2,3,4,5]. The battery replacement process is difficult and costly, when the miniaturized devices are located in remote or inaccessible locations, and practically infeasible for biomedical implantable devices. Thus, WPT is considered a cost-effective technology to power enormous devices and avoid battery replacement.

**Figure 1:**
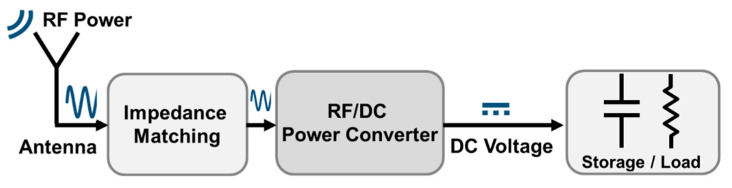
Typical block diagram of the WPT receiver.

Near-field WPT operates over short distances (i.e., less than 1 m) and delivers a few watts of power, and far-field WPT provides a radio frequency (RF) power for a longer communication distance. Far-field WPT utilizes frequency ranges from low-MHz to ultra-high frequency (UHF) or higher frequency bands. The WPT receiver consists of an antenna to capture RF energy followed by: the impedance matching network, the RF-to-DC power converter, and the storage element as shown in Figure 1. The core of the WPT receiver is the RF rectifier, which is delivered usable output DC power to the load from the input RF power incoming from the antenna. The sensitivity of the minimum input RF power required by the rectifier to generate a specific output voltage determines the wireless powering range. The power conversion efficiency (PCE), which is the ratio of the output DC power to the available input RF power from the antenna, is determined significantly by the RF-to-DC power converter. The superb wide input range of power levels is determined by the dynamic range (DR) of the RF rectifier, which operates 80% efficiently of the peak PCE [6], and the DR is given by:

(1) $$DR\;\left(\mathrm{dB}\right)=P_{max}\left(\mathrm{dBm}\right)-P_{min}\left(\mathrm{dBm}\right)$$

where $P_{min}$ and $P_{max}$ are the low- and high input power levels, where the PCE ≥ 0.8 × peak PCE. The wide *DR* enables flexibility of the distance between the transmitter and the wireless power receiver.

**Figure 2:**
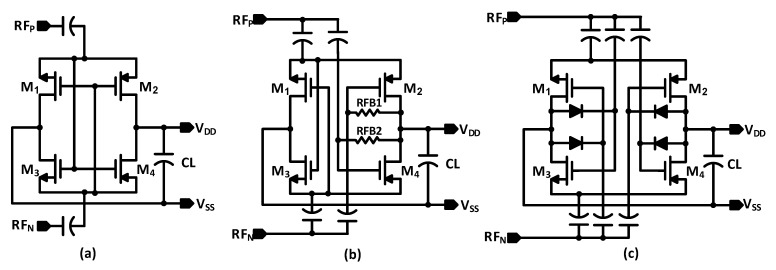
Schematic of (**a**) a conventional FX rectifier; (**b**) a self-biased rectifier; (**c**) an FB diodes rectifier.

Several works have been introduced to realize the CMOS RF-to-DC power converter. The diode-based architecture is the common RF rectifier, such as the Dickson multiplier [7]. The diodes in CMOS technology are implemented by using diode-connected transistors. However, the diode-based RF rectifier suffers from poor low power performance and high dropout voltage across the diodes, which decreases the PCE. The Schottky diodes can be used to enhance the sensitivity and the low power performance [8]; however, they are seldom offered in standard CMOS technology, and hence cannot be used in integrated systems. The fully cross-coupled differential rectifier (FX) has been employed to improve the low power performance as shown in Figure 2a [9]. It achieves high sensitivity and peak PCE. However, the FX RF rectifier achieves good efficiency in a narrow input power range due to the reverse current leakage. The rectifying transistors are bidirectional devices, unlike the diode-connected transistors. When the instantaneous input RF signal (*V_RF_*) is lower than the output voltage (*V_DD_*) in every cycle, the leakage current flows in a reverse direction and results in energy loss. The authors of [10] introduced the self-biased RF rectifier to address the leakage currents. It employs the feedback resistors to control the conductivity of the rectifying devices as shown in Figure 2b. It improves the input power range at high RF power. However, it has poor low power performance as the self-biased design reduces the forward current, which degrades the peak PCE at low RF power. Moreover, the resistors occupy a large area in silicon, and add parasitic elements, which degrade the efficiency at sub-GHz and beyond. The feedback diodes (FB) RF rectifier is introduced to address this limitation as shown in Figure 2c [11]. It achieves high PCE and improves sensitivity. Nonetheless, it suffers from the leakage currents for the NMOS rectifying devices as well as the feedback diodes, which results in a limited dynamic range. Internal threshold-voltage compensation of the rectifying transistors is introduced in [12] to increase the harvested power and reduce the leakage current; however, it suffers from poor sensitivity and limited RF input power range. The authors of [13] propose a dual-mode nested feedback circuit to lower the effective threshold voltage of the rectifier and to minimize the reverse leakage current. However, the proposed design increases the circuit complexity and parasitic elements, which degrades the performance of the rectifier at the UHF. The adaptive RF-DC converter is introduced to cover a wide input RF power range in [14], which varies the number of stages depending on the input power; however, the proposed design still has a limited dynamic range due to the reverse leakage power.

This work presents a wide-input range high-efficiency differential CMOS RF rectifier operating at a UHF band, with proposed stacking diodes for NMOS rectifying devices, and feedback diodes with an adaptive body-biasing technique for PMOS rectifying devices. The proposed novel techniques diminish the leakage currents of the rectifying transistors and the feedback diodes. The proposed design extends the high efficiency in a wide-input range of RF power levels, which allows for the efficient RF rectifier to operate at varying distances from the transmitter. The proposed architecture is introduced in Section 2, along with its design and analysis. Section 3 presents the results and discussion of the implemented prototype, and Section 4 concludes the paper.

## 2. Proposed Wide Dynamic Range RF-to-DC Power Converter

**Figure 3:**
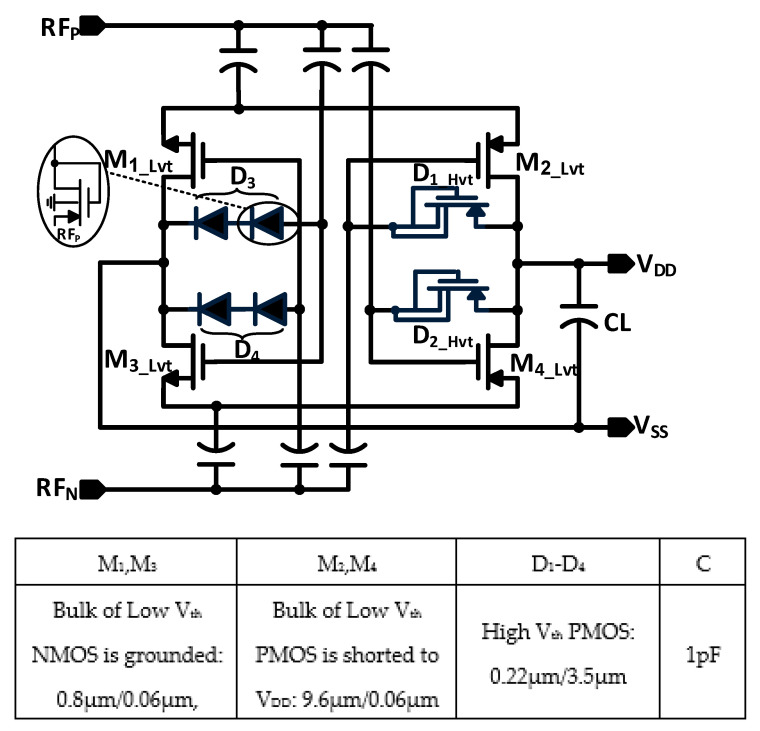
Proposed RF rectifier with adaptive biasing technique.

**Figure 4:**
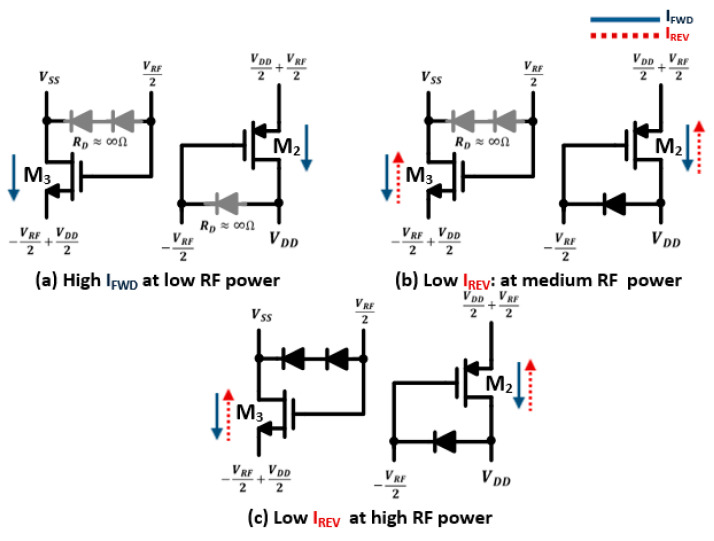
Steady-state operating points for NMOS and PMOS rectifying transistors during ON mode at (**a**) low; (**b**) medium; (**c**) high input RF power.

The conceptual schematic of the proposed RF rectifier for a wide dynamic range is shown in Figure 3. It is based on the cross-coupled differential architecture, where the rectifier has low-threshold NMOS and PMOS rectifying transistors (M_1–4_). These transistors are controlled by the stacking diodes (D_3_, D_4_) and feedback diodes (D_1_, D_2_), with dynamic body biasing and six coupling capacitors. Unlike the FB diodes rectifier, the proposed stacking diodes are implemented to form high threshold voltage in the reverse direction, where the diodes are forward biased for the RF signal and reverse biased for the DC signal. In addition, the feedback diodes are implemented using PMOS transistors, where the adaptive body biasing by the input RF signal forms dynamic threshold voltage. At low RF power levels, the diodes are turned off due to the threshold voltage being larger than the voltage drop across the diode. The proposed design is reconfigured as a cross-coupled rectifier and is driven by a differential RF input. The low threshold devices with differential drive capability have low ON-resistance and low dropout voltage, resulting in high sensitivity and PCE at low input power as they transfer high forward current to the load. Moreover, the bulk of the PMOS rectifying transistors (M_2_, M_4_) is shorted to the output DC voltage in order to lower the transistor threshold voltage at low input RF power. During the positive half cycle (RF_P_ = $\frac{V_{RF}}{2}$, RF_N_ = $-\frac{V_{RF}}{2}$) of the input RF signal, the rectifying devices (M_2_, M_3_) are turned on and operate in the linear region, as shown in Figure 4a. The forward currents (*I_FWD_*) of the *PMOS* and *NMOS* rectifying transistors (M_2_, M_3_) are given by:

(2) $$I_{FWD,PMOS}=K_{P}\;\frac{W}{L}\;\left(\left(\;V_{RF}+\frac{V_{DD}}{2}-│V_{th,P}│\right)\;V_{SD}-\frac{1}{2}\;V_{SD}{}^{2}\right)$$

and

(3) $$I_{FWD,NMOS}=K_{N}\;\frac{W}{L}\;\left(\left(\;V_{RF}-\frac{V_{DD}}{2}-V_{th,N}\right)\;V_{DS}-\frac{1}{2}\;V_{DS}{}^{2}\right)$$

where $\;K_{P}$ and $K_{N}$ are process dependent of *PMOS*/*NMOS* transistors,$\;V_{RF}$ is the instantaneous RF voltage, $V_{DD}$ is the DC voltage generated across the load, $V_{th,P}$ and $V_{th,N}$ are the threshold voltage of PMOS/NMOS transistors, $\;V_{SD}$ and $\;V_{DS}$ are source-to-drain and drain-to-source voltages, and W and  L are channel width and length, respectively. The rectifying devices (M_1_, M_4_) operate in the same way in the negative half cycle (RF_P_ = $-\frac{V_{RF}}{2}$, RF_N_ = $\frac{V_{RF}}{2}$) due to the symmetricity of the circuit. During the negative half cycle of the input RF signal, the rectifying devices (M_2_, M_3_) are turned off and operate in the weak inversion region. The leakage current ($I_{L}$), which is caused by the non-idealities of the rectifying devices (M_2_, M_3_), is given by:

(4) $$I_{L}=\left\{\begin{array}{c} I_{o}\;\frac{W}{L}\;e^{\frac{\;\left(V_{DD}-\frac{V_{RF}}{2}-│V_{th,P}│\right)}{n\;V_{thermal}}}\left(1-e^{\frac{-V_{SD}}{\;V_{thermal}}}\right)\;\mathrm{for}\;\mathrm{PMOS}\;\mathrm{device}.\\ I_{o}\;\frac{W}{L}\;e^{\frac{\;\left(-\frac{V_{RF}}{2}-V_{th,N}\right)}{n\;V_{thermal}}}\;\left(1-e^{\frac{-V_{DS}}{\;V_{thermal}}}\right)\;\mathrm{for}\;\mathrm{NMOS}\;\mathrm{device}. \end{array}\;\right.$$

where n is the subthreshold slope factor, $I_{o}$ is the process dependent parameter, and $V_{thermal}$ is the thermal voltage. The leakage current at the subthreshold region is negligible and flows in the reverse direction to the driving voltage, for $I_{L}$ is less than the threshold voltage of the rectifying devices.

At medium RF power levels the output DC voltage is increased, and the instantaneous RF input voltage at the source of the PMOS rectifying devices becomes less than $V_{DD}$. Therefore, it is critical to minimize the reverse leakage current while the rectifying transistors are turned on as they are bilateral devices. Therefore, the proposed design utilizes the feedback diodes (D_1_, D_2_) implemented using PMOS transistors with an adaptive body biasing technique as shown in Figure 4b. The body voltage of the diode is tied to the RF input voltage, which forms adaptive threshold voltages. During the positive half-cycle of the input RF voltage, the diode (D_1_) is turned on when the voltage across it is larger than the threshold voltage of the diode ($V_{th,D}$), which is decreased due to lowering the body voltage (i.e., $\left(-V_{RF}/2\right)<(V_{DD}-V_{th,D})$). Thus, the diode is changed to a short circuit with a low dropout voltage, and this lowers the driving voltage of the PMOS rectifying transistor by changing its DC operating points. The DC voltage at the gate of the PMOS rectifying transistor ($V_{DC}$) is given by

(5) $$V_{DC}=\frac{1}{2C_{eq}}\int \;K_{P}\;\frac{W}{L}{\left(\;V_{DD}+\frac{V_{RF}}{2}-│V_{th,D}│\right)}^{2}dt$$

where $C_{eq}$ is the equivalent capacitance seen at the gate of the PMOS rectifying transistor. Therefore, the reverse leakage current ($I_{REV,PMOS}$) of the rectifying device is minimized, which is given by:

(6) $$I_{REV,PMOS}=K_{P}\;\frac{W}{L}\;\left(\left(V_{DD}+\frac{V_{RF}}{2}-V_{DC}-│V_{th,P}│\right)\;V_{SD}-\frac{1}{2}\;V_{SD}{}^{2}\right)$$

The minimized $I_{REV,PMOS}$ enhances the PCE performance of the rectifier. During the negative half-cycle of the input RF voltage, the feedback diode (D_1_) is turned off as the $V_{RF}/2$ is greater than $\left(V_{DD}-V_{th,D}\right)$. The rising of the RF input voltage increases the body voltage of the diode-connected transistor (D_1_), which increases the $V_{th,D}$. Therefore, it decreases the leakage current of the feedback diode at medium and high RF power levels.

**Figure 5:**
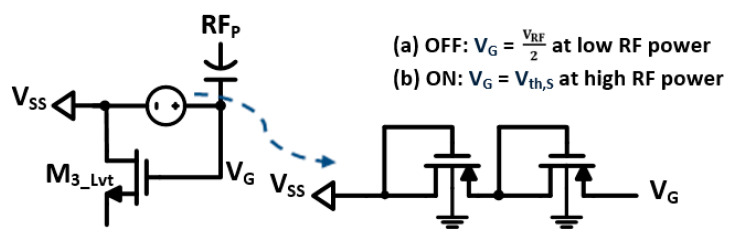
Adaptive biasing technique for the NMOS rectifying transistors.

On the other hand, the reverse leakage current of the low threshold NMOS rectifying transistors is exacerbated at high RF power levels. The proposed design utilizes a new technique to set adaptive bias voltages on the gates of the NMOS rectifying devices in order to control their conduction. The adaptive bias voltage is implemented, using the weak conduction stacking diodes (D_3_, D_4_), to form a high threshold voltage and minimize the reverse leakage current. Therefore, the diode’s operation is limited at high RF power levels, as shown in Figure 4c. The diodes are forward biased when the voltage across them is larger than the threshold voltage of the stacking diodes. In addition, the body of the diodes is grounded to produce dynamic bulk-source voltages, and hence provides adaptive bias voltage, as shown in Figure 5. Thus, the driving voltage is reduced and set by the threshold voltage of the stacking diodes ($V_{th,S})$; as the RF voltage grows, the $V_{th,S}$ decreases. Thus, the reverse leakage current for the NMOS devices is reduced and can be expressed by:

(7) $$I_{REV,NMOS}=K_{N}\;\frac{W}{L}\;\left(\left(V_{th,S}-V_{th,N}\right)\;V_{DS}-\frac{1}{2}\;V_{DS}{}^{2}\right)$$

and $V_{th,S}$ is given by

(8) $$V_{th,S}=│2V_{th0}+\gamma \;\left(\left(-2\sqrt{│2\phi_{F}│}\right)+\sqrt{│\left(-2\right)\phi_{F}+\frac{V_{RF}}{2}│}+\sqrt{│\left(-2\right)\phi_{F}+\frac{V_{RF}}{2}-V_{SG,1}}│\right)│$$

where γ is the body effect coefficient, $\phi_{F}$ is Fermi potential, $V_{th0}$ is the initial threshold voltage, and $V_{SG,1}$ is the source gate voltage [15]. Thus, the conductivity of the NMOS rectifying devices is decreased from $\left(\frac{V_{RF}}{2}-V_{th,N}\right)$ at low RF power to $\left(V_{th,S}-V_{th,N}\right)$ at high RF power to limit the reverse leakage power, and the minimized reverse current at high RF power levels improves the high-power performance of the proposed RF rectifier, which extends the dynamic range operating at high efficiency from low to high power levels. 

## 3. Results and Discussion

**Figure 6:**
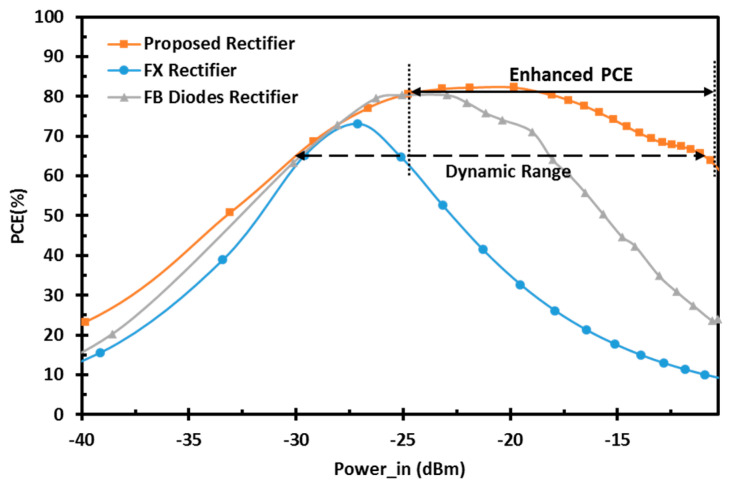
Simulated PCE for conventional FX and FB diodes, and proposed rectifiers vs. the input RF power.

**Figure 7:**
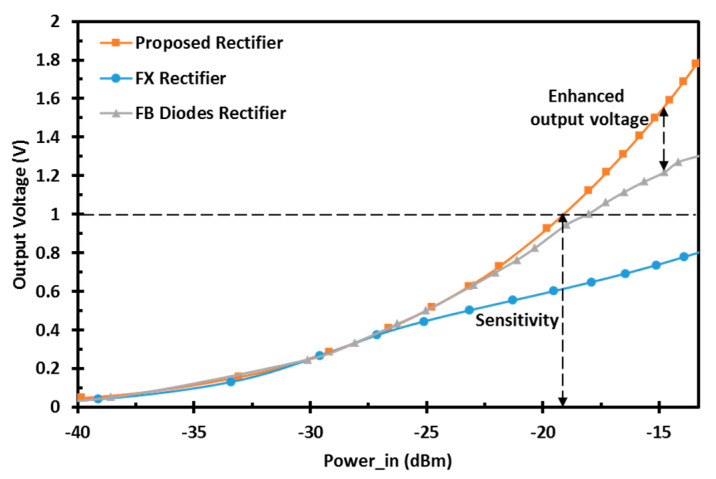
Simulated output DC voltage vs. input RF power for different architectures at a UHF 900 MHz band with a 100-KΩ load.

**Figure 8:**
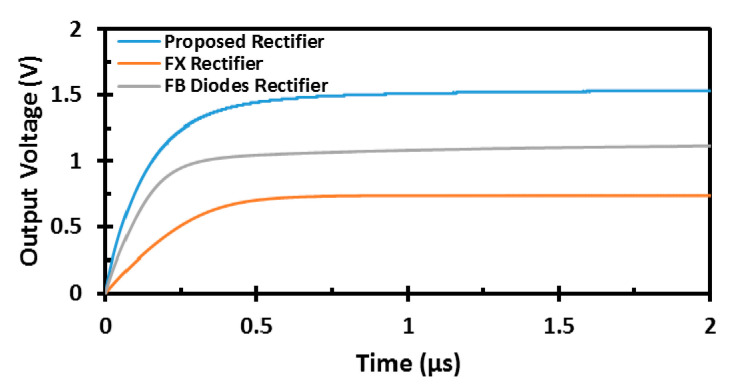
Transient simulation of the output DC voltage for the different architectures at −15 dBm input RF power level for a load capacitance of 0.1 nF.

The proposed architecture is implemented and simulated using 65 nm standard CMOS technology. For a fair comparison, Figure 6 demonstrates the proposed design performance with the conventional FX and FB diodes rectifiers at the same technology. It shows the PCE versus the input RF power operating at a UHF 900 MHz band with a 100-KΩ load. The PCE is calculated by

(9) $$PCE=\frac{P_{out}}{P_{in}}=\frac{V_{DD}^{2}}{P_{in}R_{load}}$$

where $P_{out}$ is the output DC power, and $P_{in}$ is the input power delivered to the rectifier by de-embedding the losses of the transmission and the reflection [10,11]. The proposed rectifier offers the highest peak PCE of 82.2% and maintains 80% of its peak PCE from the low to high power levels, which corresponds to a DR of 18.45 dB. The proposed design has 17 times better DR than the conventional FX rectifier and 5.7 times better DR than the FB diodes rectifier in 65 nm CMOS technology. In addition, the sensitivity of the proposed design is shown in Figure 7. The proposed design achieves the best sensitivity of −19.1 dBm for 1 V output DC voltage compared with the different architectures and enhances the output DC voltage at high power levels, allowing it to reach the same output voltage at a lower input RF power. Figure 8 shows the transient simulation of the output DC voltage for the different architectures at high RF power levels. The proposed design achieves better output DC voltage at −15 dBm input RF power level with a 100-KΩ load for a load capacitance of 0.1 nF.

**Figure 9:**
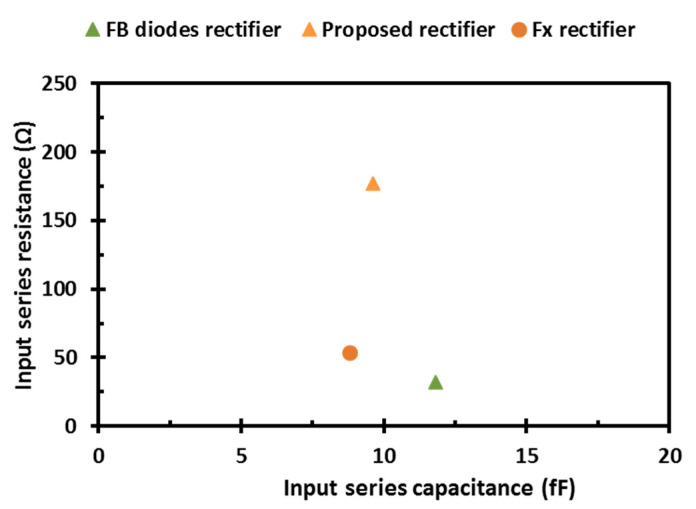
Input impedance of the proposed rectifier with the conventional FX and FB diodes rectifiers.

Figure 9 shows the input impedance of the proposed rectifier with the different architectures. The proposed design has an input series resistance and capacitance of 177.2 Ω and 9.6 fF, respectively. The matching network can be implemented to match the input impedance of the rectifier to the designed antenna for the largest total efficiency.

**Figure 10:**
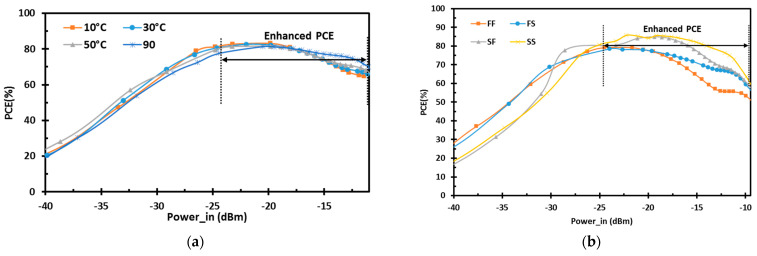
Simulation results showing the variation of the PCE for the proposed rectifier vs. input RF power at (**a**) different temperature levels; (**b**) process corners.

Figure 10a shows the variation of the PCE of the proposed design for the temperature range from 10 °C to 90 °C. The proposed design has low temperature sensitivity and maintains the enhanced efficient operating region from the low- to the high-power levels. The PCE of the proposed rectifier at different process corners is shown in Figure 10b. The process variations are covered by the process corner simulations at fast–fast (FF), fast–slow (FS), slow–fast (SF), and slow–slow (SS) corners. The proposed design keeps the enhanced low and high power performances with excellent DR.

**Figure 11:**
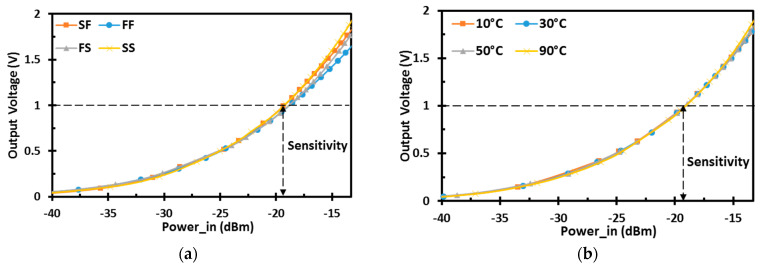
Output DC voltage of the proposed rectifier: (**a**) at different process corners; (**b**) at different temperature levels.

Figure 11 shows the output DC voltage versus the input RF power at different process corners and temperature levels. The proposed design maintains high sensitivity for 1 V output voltage, operating at 900 MHz with a 100-KΩ load.

**Figure 12:**
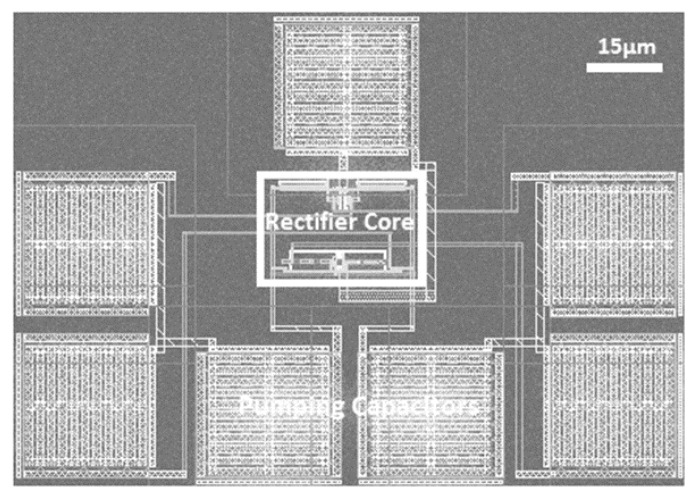
Chip layout of the RF CMOS rectifier prototype.

**Figure 13:**
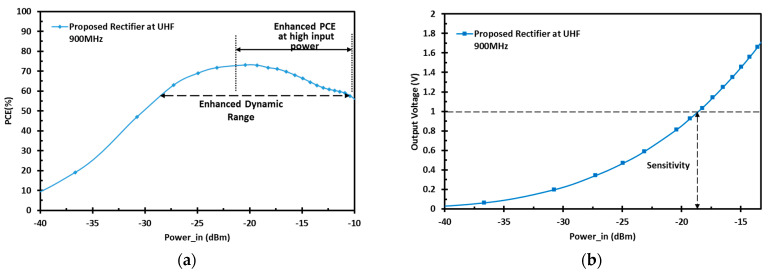
Post-layout simulation results of (**a**) the PCE vs. input RF power; (**b**) output DC voltage vs. input RF power.

**Figure 14:**
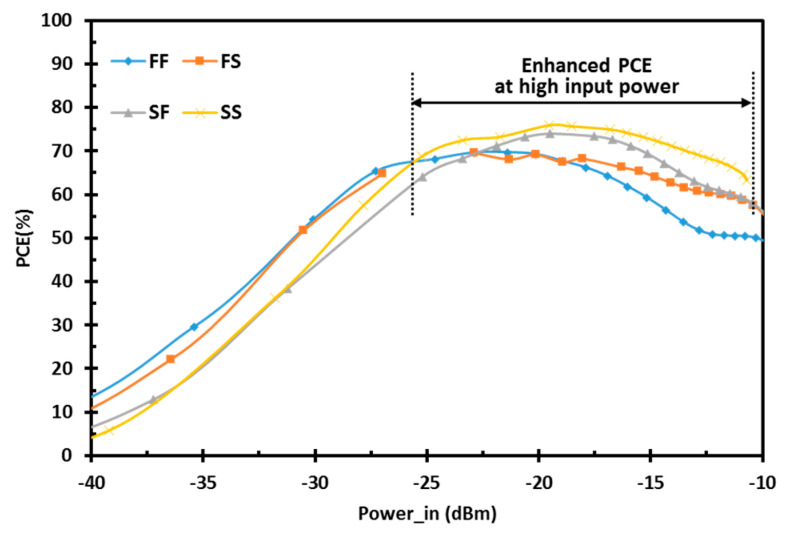
Post-layout simulation results of the PCE vs. input RF power at different process corners.

**Figure 15:**
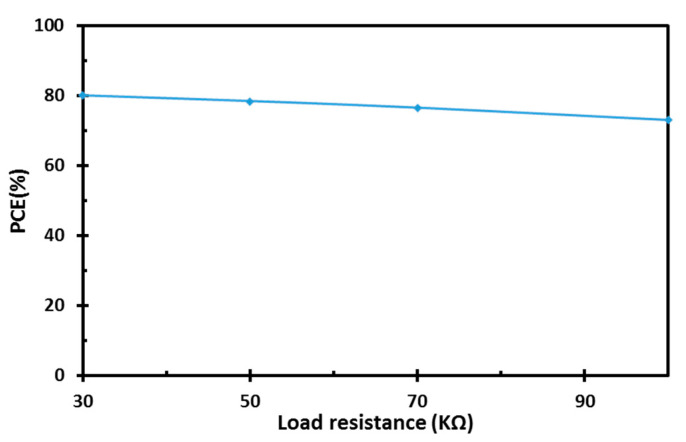
Post-layout simulation result of the peak PCE for different resistive loads.

The RF rectifier prototype is implemented and laid out using the 65 nm CMOS technology, as shown in Figure 12. The silicon chip occupies an active area of 0.0093 mm^2^ and it includes the pumping capacitors. The post-layout simulation results of the proposed design, including the parasitic elements at a UHF 900 MHz band with a 100-KΩ load, are shown in Figure 13. The proposed design exhibits superb PCE with a wide-input power range, as shown in Figure 13a. The achieved peak PCE is 73%, and the proposed design maintains 80% of its peak PCE across the low- and high-power levels, which corresponds to a DR of 17.3 dB. Moreover, Figure 13b shows the output DC voltage versus the input RF power. The post-layout simulation result shows that the proposed design achieves high sensitivity of −18.8 dBm to generate 1 V across the load. The PCE under process variations is shown in Figure 14. The proposed design offers enhanced power conversion efficiency at different process corners. Figure 15 shows the post-layout simulation of the peak PCE at different resistive loads. The proposed design shows an insignificant change in peak PCE, and improvement in the performance for different loads is maintained.

**Figure 16:**
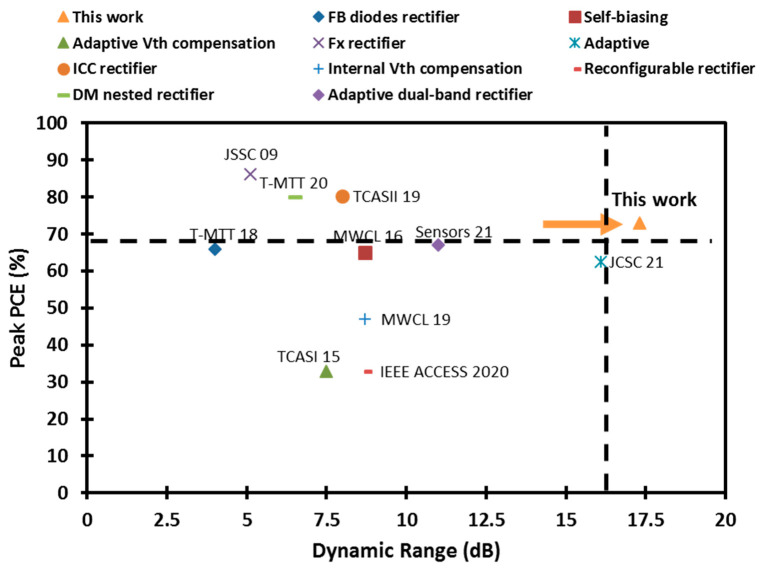
Peak PCE vs. the dynamic range of the published RF CMOS rectifiers at the UHF band.

Table 1 compares the performance of the proposed architecture with the state-of-the-art RF CMOS rectifiers at a similar harvesting UHF band. To the best of the author’s knowledge, the proposed rectifier achieves the best DR, sensitivity, and low power performance reported to date, as shown in Table 1. It achieves a competitive DR of 17.3 dB, which is 12.2 dB wider than the conventional FX rectifier [9], 13.3 dB wider than the FB diodes rectifier [11], and 8.6 dB wider than the self-biasing rectifier [16] due to the superb low- and high-power performances. It has a compact and small silicon area of 0.0093 mm^2^. In addition, it has a better sensitivity and dynamic range than the voltagethreshold-compensated, DM nested, and adaptive rectifiers [12,13,14]. Compared to the low power performance, the proposed design offers the best peak PCE at −35 dBm of 25.5%. Figure 16 shows the peak PCE versus the DR of recently published architectures. The proposed design is the first-reported rectifier to cross the 17 dB DR boundary with an efficiency higher than 70% at the UHF band.

## 4. Conclusions

A new wide-dynamic range RF CMOS rectifier with high efficiency has been proposed at the UHF band. The proposed rectifier employs the adaptive-biasing scheme for the NMOS rectifying transistors to increase the forward current at a low-input power, and to minimize the leakage current at high-RF power levels. The adaptive-biasing scheme is implemented using the stacking diodes, with an adaptive threshold voltage that is reduced at high-input power; therefore, it decreases the driving voltage of the NMOS rectifying transistors. The proposed design also utilizes self-bulk biasing for the feedback diodes to reduce its leakage current and to minimize the conductivity of the PMOS rectifying transistors at high power levels. The achieved DR of the proposed design in the 65 nm CMOS technology is 17.3 dB, which is the best DR with a high peak PCE of 73% reported to date. The proposed rectifier offers the best low power performance with a high peak PCE at −35 dBm of 25.5% and a high sensitivity of −18.8 dBm.

## Figures and Tables

**Table 1:** Comparison with state-of-the-art RF CMOS rectifiers at the UHF Band.

Architecture	Technology	Area (mm^2^)	Load (KΩ)	Frequency (MHz)	PCE at −35 dBm (%)	Peak PCE (%)	Dynamic Range ^a^ (dB)	Sensitivity ^b^ (dBm)
Proposed	65 nm	0.0093	100	900	25.5	73	17.3	−18.8
Fx rectifier [9]	180 nm	0.0134	100	953	<18	86.2	5.1	−12.9
FB diodes rectifier [11]	180 nm	0.0088	100	900	<8	66	4	−18.2
Internal *V_th_* compensation [12]	180 nm	0.1	5	902	<1	47	8.7	−3
DM nested rectifier [13]	65 nm	0.0064	100	900	19	80	6.5	−14.9
Adaptive dual band RF-DC converter [14]	180 nm	0.115	100	900/2400	<5	67.1	11	−17
Self-biasing [16]	180 nm	0.0084	100	900	<10	65	8.7	−18
Adaptive *V_th_* compensated [17]	130 nm	0.25	130	915	N/A	33	7.5	−17
Adaptive [18]	65 nm	0.0148	100	900	11	62.6	16.1	−17.27
ICC rectifier [19]	130 nm	0.062	100	900	<1	80.3	8	−18.7
Reconfigurable rectifier [20]	180 nm	0.1	200	902	N/A	33	8.7	−16.9

^a^ input RF power range of PCE > 0.8 × peak PCE; ^b^ input RF power for 1 V output voltage.
